# Enantiospecific synthesis of [2.2]paracyclophane-4-thiol and derivatives

**DOI:** 10.3762/bjoc.5.9

**Published:** 2009-03-12

**Authors:** Gareth J Rowlands, Richard J Seacome

**Affiliations:** 1 Institute of Fundamental Sciences, Massey University, Private Bag 11 222, Palmerston North, New Zealand; 2 Chemistry Division, Department of Chemistry and Biochemistry, University of Sussex, Falmer, Brighton, BN1 9QJ, UK

**Keywords:** heterocycle, [2.2]paracyclophane, resolution, sulfur

## Abstract

This paper describes a simple route to enantiomerically enriched [2.2]paracyclophane-4-thiol via the stereospecific introduction of a chiral sulfoxide to the [2.2]paracyclophane skeleton. The first synthesis of an enantiomerically enriched planar chiral benzothiazole is also reported.

## Introduction

**Figure 1:**
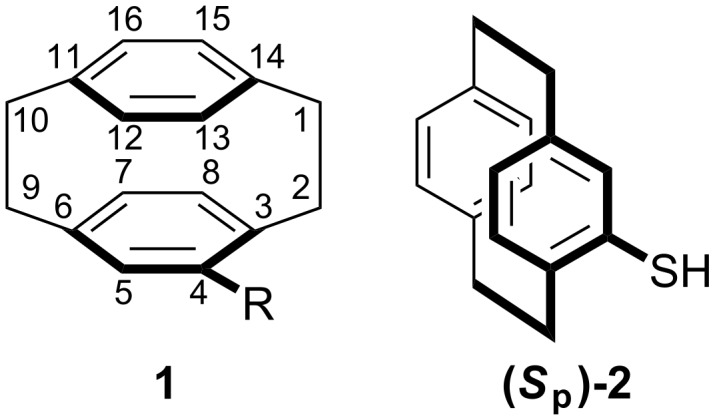
[2.2]Paracyclophane (**1**) showing standard numbering and [2.2]paracyclophane-4-thiol (**2**).

[2.2]Paracyclophane (**1**; R = H) is a fascinating compound comprising of two eclipsing benzene rings that are held in place by two ethyl bridges at the para positions (Figure 1). The close proximity of the arene moieties results in strong electronic and structural interactions between the two rings and between substituents appended to each layer [1–2]. The resulting unique properties have led to derivatives of [2.2]paracyclophane being employed in a wide range of disciplines including polymer, material and electronic chemistry [3–9]. Whilst enantiomerically pure derivatives have been utilised in chiral catalysis [10–11] and as probes for biological recognition processes [12–14], the full potential of these systems has not been realised due to the difficulties encountered when trying to access enantiomerically pure [2.2]paracyclophane derivatives [15].

Chiral sulfur [2.2]paracyclophane derivatives are beginning to attract attention due to the great potential such compounds exhibit [16–18]. Non-cyclophane-based thiophenol derivatives have been employed in the nucleophilic addition of thioacetals to suitable electrophiles [19–20], sigmatropic rearrangements [21] and as either thiyl radical precursors [22] or as a source of hydrogen in radical chemistry [23]. With the appropriate sulfur derivative, stereoselective variants of all these transformations can be envisaged.

Currently, there are few examples of sulfur containing [2.2]paracyclophane compounds; aryl sulfonylation and the related sulfenylation facilitates the synthesis of sulfonic acids, sulfonamides and protected thiols [24–26] whilst directed metallation has allowed the formation of various sulfides [27–30]. Very few methodologies allow the synthesis of simple chiral monosubstituted thiols such as [2.2]paracyclophane-4-thiol **2** (Figure 1); the first reported preparations of racemic **2** were the conversion of 4-hydroxy[2.2]paracyclophane to the desired compound *via* a Newman-Kwart reaction or the direct reaction of 4-lithio[2.2]paracyclophane with sulfur [17]. Use of enantiomerically pure 4-hydroxy[2.2]paracyclophane or application of our own sulfoxide-metal exchange protocol [31] would permit enantiospecific variants of either route, but neither has been reported. An elegant entry to a variety of racemic alkyl sulfides and sulfoxides by an S_E_Ar reaction mediated by a sulfonium salt has recently been divulged [16]. The only reported synthesis of enantiomerically pure [2.2]paracyclophane-4-thiol entails the palladium-mediated addition of triisopropylsilanethiol to a triflate formed from previously resolved (*R*)-4-hydroxy[2.2]paracyclophane [18]. We are developing a ‘tool-box’ for the synthesis of enantiomerically enriched [2.2]paracyclophane derivatives based on the chemistry of [2.2]paracyclophane sulfoxides [15,31–33]. This methodology has allowed us to develop routes to enantiomerically pure 4-monosubstituted [2.2]paracyclophanes [31] along with a range of disubstituted derivatives [33]. The basis of the strategy is the stereospecific introduction of a sulfoxide to [2.2]paracyclophane to give readily separable diastereoisomers, thus resolving the planar chirality [34]. The sulfoxide moiety is used to direct further elaboration of the [2.2]paracyclophane framework or is displaced *via* sulfoxide-metal exchange. We were interested in modifying this methodology to permit the synthesis of enantiomerically enriched [2.2]paracyclophane-4-thiol and related compounds.

## Results and Discussion

**Scheme 1:**
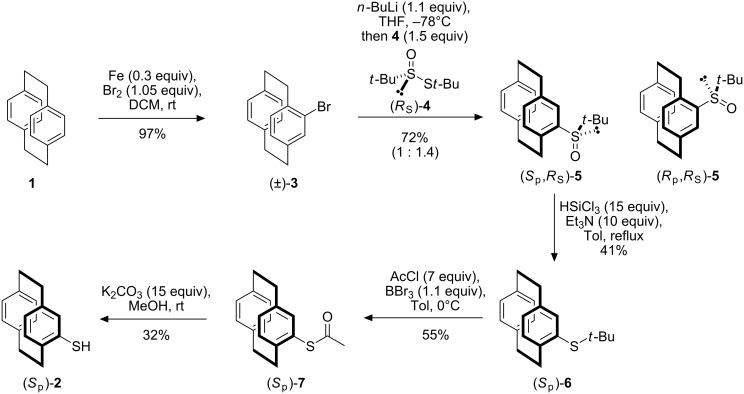
Conversion of [2.2]paracyclophane to enantiomerically enriched [2.2]paracyclophane-4-thiol.

The synthesis of (*S*_p_)-**2** is depicted in Scheme 1. Key to the success of this strategy was the resolution of the planar chirality of [2.2]paracyclophane by incorporation of the *tert*-butylsulfinyl moiety to give the diastereoisomers (*S*_p_,*R*_S_)-**5** and (*R*_p_,*R*_S_)-**5**. Standard iron-catalysed bromination of **1** gave (±)-4-bromo[2.2]paracyclophane **3** in good yield [35–36]. Halogen-lithium exchange and addition to Ellman’s (*R*)-*tert*-butyl *tert*-butanethiosulfinate [37] **4** furnished a 1 : 1.4 mixture of (*S*_p_,*R*_S_)-**5** and (*R*_p_,*R*_S_)-**5** in a combined 72% yield [33]. The two diastereoisomers are readily separable by standard column chromatography, with the diastereoisomer (*S*_p_,*R*_S_)-**5** being eluted first. As (*R*)-**4** was prepared with an *ee* of 80%, as judged by optical rotation, we assume that each diastereoisomer displays an *ee* of 80%. As the sulfinylation reaction proceeds with inversion at sulfur, the two diastereoisomers only differ by the chirality of the [2.2]paracyclophane therefore allowing the facile resolution of the planar chirality. The assignment of configuration is based on a combination of X-ray studies [33,38], formation of all stereoisomers and analogy to our previous tolylsulfinyl chemistry [31,39].

Unlike the previously prepared 4-tolylsulfinyl[2.2]paracyclophane [31], direct sulfoxide-metal exchange was not possible with the *tert*-butyl derivative [38]; presumably, the *tert*-butyl group and the lower ring of the [2.2]paracyclophane moiety shield the sulfur from attack. As a result a stepwise procedure for the conversion of (*S*_p_,*R*_S_)-**5** to [2.2]paracyclophane-4-thiol (*S*_p_)-**2** was investigated (Scheme 1). The first step, the reduction of (*S*_p_,*R*_S_)-**5** to sulfide (*S*_p_)-**6**, proved the most problematic; use of a large excess of trichlorosilane and triethylamine resulted in deoxygenation in moderate yield after recrystallisation [40]. By comparison, reduction of the less hindered aryl sulfoxide, (*R*_p_,*S*_S_)-4-bromo-13-*p*-tolylsulfinyl[2.2]paracyclophane, occurs efficiently in 98% yield suggesting the *tert*-butyl group is the source of the problem [39]. Exchange of the *tert*-butyl group for an acetyl group was achieved by reaction of a mixture of (*S*_p_)-**6** and acetyl chloride in toluene with boron tribromide [41]. The resulting thioacetic acid *S*-[2.2]paracyclophane ester (*S*_p_)-**7** readily undergoes simple base-catalysed hydrolysis to give the desired (*S*)-(+)-[2.2]paracyclophane-4-thiol (*S*_p_)-**2**.

**Scheme 2:**
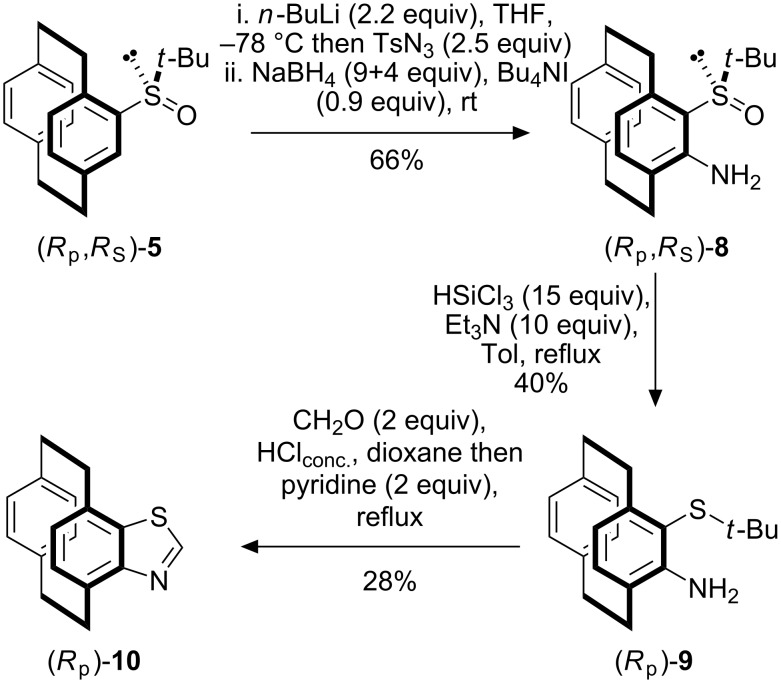
Synthesis of [2.2](4,7)benzo[*d*]thiazoloparacyclophane (*R*_p_)-**10**.

There are two advantages to our methodology compared to the previously reported syntheses of [2.2]paracyclophane thiols; the first is that resolution of the planar chirality is complicit in the addition of the sulfur moiety and does not require resolution of any precursors. Secondly, the sulfinyl moiety permits further functionalisation of the [2.2]paracyclophane skeleton [33]. It is the latter reason that prompted the synthesis of the thiol *via* the *tert*-butyl derivative and not by direct sulfoxide-metal exchange; whilst this route would have delivered **2** more rapidly it would not have permitted elaboration of the [2.2]paracyclophane framework. The utility of the *tert*-butyl derivative is demonstrated in the synthesis of the planar chiral benzothiazole (*R*_p_)-**10** (Scheme 2).

Benzothiazoles are important heterocycles having found use as dyes, pharmaceuticals and ligands in catalysis [42]. Planar chiral heterocycles are still rare but show considerable potential as probes in stereocontrolled recognition processes in biological systems as highlighted by Gmeiner [12–14] and as ligands or catalysts [43–47]. We have previously prepared planar chiral benzimidazoles [32] and wanted to extend the range of heterocycles that could be accessed.

Diastereoisomer (*R*_p_,*R*_S_)-**5** was functionalised by sulfinyl-directed *ortho* lithiation with *n*-butyllithium followed by reaction with tosyl azide. The resulting azo[2.2]paracyclophane was reduced *in situ* to give the amine (*R*_p_,*R*_S_)-**8** in good yield for the two steps (Scheme 2). Trichlorosilane-mediated deoxygenation proceeded uneventfully to furnished amino sulfide (*R*_p_)-**9**. Simultaneous sulfide deprotection and thiazole formation was achieved by treating (*R*_p_)-**9** with concentrated hydrochloric acid, paraformaldehyde and pyridine [48]. Although the yield of (*R*_p_)-**10** is not yet satisfactory, it shows the potential of our methodology for the formation of these valuable heterocycles.

In conclusion, we have developed a straightforward method for the synthesis of enantiomerically enriched [2.2]paracyclophane-4-thiol that does not rely on the resolution of precursors to the introduction of the sulfur moiety. Furthermore, we have shown that this methodology has the potential to produce a wide-range of thiol derivatives and this has permitted the first synthesis of a planar chiral benzothiazole, [2.2](4,7)benzo[*d*]thiazoloparacyclophane. The use of these thiols in asymmetric synthesis is currently being investigated and will be reported in due course.

## Experimental

NMR spectra were recorded on a Bruker 400 MHz , Bruker 300 MHz, Varian 500 MHz or Varian 400 MHz using residual isotopic solvent as internal reference. Infrared spectra were recorded on a Perkin-Elmer 1600 Fourier Transform spectrometer. Mass spectra and exact mass data were recorded by Dr. Ali Abdul-Sada at the University of Sussex or by the EPSRC national mass spectrometry service, Swansea. Melting points were recorded on a Gallenkamp melting point apparatus and are uncorrected. Optical rotation was recorded on a Perkin Elmer 241 polarimeter using a sodium lamp emitting at 589 nm. All samples were measured in chloroform (*c* = 1) in a 10 cm cell and an average taken of 10 readings; average temperature was 27 °C. Glassware was oven dried and reactions were performed under an inert atmosphere of nitrogen or argon where applicable. Chromatography refers to flash column chromatography on Merck Kieselgel 60 (230-400 mesh) or Fischer Davisil 60 silica gel unless otherwise stated. TLC refers to analytical thin-layer chromatography performed using pre-coated glass-backed plates (Merck Kieselgel 60 F_254_) and visualised with ultraviolet light, iodine, acidic ammonium molybdate (IV), acidic ethanolic vanillin, aqueous potassium manganate(VII), ninhydrin or acidic anisaldehyde as appropriate. Petrol refers to redistilled petroleum ether (60–80 °C), and ether to diethyl ether. Ether and THF were distilled from sodium-benzophenone ketyl, toluene from 4Å molecular sieves or calcium chloride. Dioxane was stored over sodium wire and DMF was stored over 4Å molecular sieves.

### (±)-4-Bromo[2.2]paracyclophane (**3**)

All stages of this reaction were performed in the dark by covering the flasks with aluminium foil. Bromine (7.8 mL, 0.15 mol, 1.05 equiv) was dissolved in DCM (1.5 L). 10% of the solution (150 mL) was transferred to a flask containing iron filings (2.4 g, 0.04 mol, 0.3 equiv) and stirred at rt for 1.5 h. A solution of [2.2]paracyclophane (30.0 g, 0.14 mol, 1.0 equiv) in DCM (2.8 L) was added and the suspension stirred for 20 min. The remaining solution of bromine in DCM (1350 mL) was added via cannula over 30 minutes. After 5 min TLC indicated complete reaction and saturated aqueous NH_4_Cl solution (5.0 L) was added. The aqueous phase was extracted with DCM (3 × 1 L) and the combined organic extracts washed with aqueous Na_2_S_2_O_3_ solution (10% w/v; 1 L) then dried (MgSO_4_). The solvent was removed to give **3** as a white powder (40.2 g, 97%); mp = 134–135 °C (lit. [35]: 136–138 °C); ν_max_ (film) 3055, 2987, 1422, 1265, 896, 739 and 705 cm^−1^; δ_H_ (300 MHz, CDCl_3_) 7.15 (1H, d, *J* = 9.0 Hz, H-13), 6.56 (1H, d, *J* = 9.0 Hz, H-8), 6.51–6.46 (4H, m, H-5, H-7, H-15 and H-16), 6.44 (1H, d, *J* = 9.0 Hz, H-12), 3.46 (1H, ddd, *J* = 10.2, 7.7, 2.1 Hz, H-2 *endo*), 3.24–3.15 (1H, m, H-1 *endo*), 3.98–3.13 (5H, m, H-1 *exo*, 2 × H-9 and 2 × H-10), 2.95–2.77 (1H, m, H-2 *exo*); δ_C_ (75 MHz, CDCl_3_) 142.0, 139.7, 139.5, 137.6, 135.4, 133.7, 133.4, 133.3, 132.6, 131.8, 129.1, 127.4, 36.2, 35.9, 35.2, 33.8.

### (*R*_p_,*R*_S_)-(–)-4-*tert*-Butylsulfinyl[2.2]paracyclophane [(*R*_p_,*R*_S_)-**5**] and (*S*_p_,*R*_S_)-(–)-4-*tert*-Butylsulfinyl[2.2]paracyclophane [(*S*_p_,*R*_S_)-**5**]

To a solution of (±)-4-bromo[2.2]paracyclophane (5.50 g, 19.16 mmol, 1.0 equiv) in THF (180 mL) at −78 °C was added *n*-BuLi (2.5 M in hexanes; 8.5 mL, 21.08 mmol, 1.1 equiv) dropwise over 15 min. After 45 min, (*R*)-*tert*-butyl *tert*-butanethiosulfinate (*R*)-**4** (80% *ee*; 5.57 g, 28.74 mmol, 1.5 equiv) was added as a solid and the reaction stirred at rt overnight. The solvent was removed and the resulting residue purified by chromatography (Et_2_O/*n*-heptane gradient) to yield (*R*_p_,*R*_S_)-**5** (1.79 g, 30%) and (*S*_p_,*R*_S_)-**5** (2.51 g, 42%).

#### (*R*_p_,*R*_S_)-(–)-4-*tert*-Butylsulfinyl[2.2]paracyclophane [(*R*_p_,*R*_S_)-5]

mp 124–126 °C; [α]_D_ −39.6 (*c* 1, CHCl_3_) (assumed 80% *ee* see text); ν_max_ (film) 2962, 2926, 1585, 1456, 1473, 1500, 1170, 1054, 1024, 908 and 847 cm^−1^; δ_H_ (500 MHz, CDCl_3_) 7.02 (1H, s, H-5), 6.83 (1H, d, *J* = 7.5 Hz, H-13), 6.62 (1H, d, *J* = 7.5 Hz, H-7), 6.54 (1H, d, *J* = 8.0 Hz, H-12), 6.52 (2H, s, H-15, H-16), 6.48 (1H, d, *J* = 8.0 Hz, H-8), 3.54 (1H, ddd, *J* = 13.5, 12.3, 2.5 Hz, H-2 *endo*), 3.27 (1H, ddd, *J* = 13.0, 9.1, 5.5 Hz, H-1 *endo*), 3.16–3.06 (5H, m, H-1 *exo*, 2 × H-9 & 2 × H-10), 2.89 (1H, ddd, *J* = 10.0, 8.9, 5.5 Hz, H-2 *exo*), 1.05 (9H, s, *t*-Bu); δ_C_ (125 MHz, CDCl_3_) 140.7 (C), 139.5 (C), 139.0 (C), 138.9 (C), 138.9 (C), 136.0 (CH), 134.6 (CH), 133.1 (CH), 132.7 (CH), 132.6 (CH), 132.3 (CH), 130.3 (CH), 56.6 (C), 35.2 (CH_2_), 35.1 (CH_2_), 34.7 (CH_2_), 33.6 (CH_2_), 22.7 (CH_3_); *m/z* (EI+) 256 [M-*t*-Bu]^+^, 240, 152, 135, 123, 104, 91, 78 (Found: [M]^+^, 312.1539. C_20_H_24_OS requires [M]^+^, 312.1542).

#### (*S*_p_,*R*_S_)-(+)-4-*tert*-Butylsulfinyl[2.2]paracyclophane [(*S*_p_,*R*_S_)-5]

mp = 122–124 °C; [α]_D_ +151.4 (*c* 1, CHCl_3_) (assumed 80% *ee* see text); ν_max_ (film) 2970, 2927, 2852, 1587, 1474, 1459, 1432, 1410, 1175, 1039, 904, 847 and 805 cm^−1^; δ_H_ (500 MHz, CDCl_3_) 6.93 (1H, d, *J* = 10.5 Hz, H-13), 6.58 (1H, d, *J* = 10.0 Hz, H-12), 6.54–6.47 (5H, m, H-5, H-7, H-8, H-15, H-16), 4.35 (1H, t, *J* = 14.5 Hz, H-2 *endo*), 3.37 (1H, ddd, *J* = 12.5, 13.0, 7.0 Hz, H-1 *endo*), 3.22–2.98 (5H, m, H-1 *exo*, 2 × H-9 and 2 × H-10), 2.82–2.77 (1H, m, H-2 *exo*), 1.05 (9H, s, *t*-Bu); δ_C_ (125 MHz, CDCl_3_) 142.2 (C), 140.7 (C), 139.3 (C), 139.0 (C), 137.7 (CH), 135.7 (CH), 134.2 (CH), 133.3 (CH), 133.0 (CH), 132.7 (CH), 132.6 (CH), 132.5 (CH), 56.5 (C), 36.1 (CH_2_), 35.2 (CH_2_), 35.0 (CH_2_), 34.2 (CH_2_), 23.1 (CH_3_); *m/z* (EI+) 256 [M − *t*-Bu]^+^, 240, 207 [[2.2]paracyclophane]^+^, 152, 136, 123, 104, 91, 78 (Found: [M]^+^, 312.1545. C_20_H_24_OS requires [M]^+^, 312.1542).

### (*S*_p_)-(+)-4-*tert*-Butylsulfanyl[2.2]paracyclophane [(*S*_p_)-**6**]

Triethylamine (11.92 mL, 85.58 mmol, 10 equiv) was added to a solution of trichlorosilane (17.9 mL, 128.36 mmol, 15 equiv) and (*S*_p_,*R*_S_)-(+)-4-*tert*-butylsulfinyl[2.2]paracyclophane (2.67 g, 8.56 mmol, 1.0 equiv) in toluene (41 mL). The reaction was heated to reflux for 18 hours. After cooling to 0 °C a solution of aqueous NaOH (3.0 M; 200 mL) was added carefully. The aqueous phase was extracted with Et_2_O (3 × 100 mL) and the combined organic phases dried (MgSO_4_). After removal of the solvent, the residue was purified by chromatography (5% Et_2_O/hexane) followed by trituration of the yellow semi-solid with petrol gave (*S*_p_)-**6** as a white solid (1.0 g, 41.0%); mp = 53 °C; [α]_D_ + 89.5 (*c* 1, CHCl_3_) (assumed 80% *ee* see text); ν_max_ (film) 2957, 2926, 2894, 1471, 1455, 1433, 1411, 1389, 1362 and 1165 cm^−1^; δ_H_ (500 MHz, CDCl_3_) 6.68 (1H, s, H-5), 6.68 (1H, d, *J* = 10.0 Hz, H-13), 6.54 (2H, dd, *J* = 8.0, 3.5 Hz, H-7, H-8), 6.50 (2H, s, H-15, H-16), 6.45 (1H, d, *J* = 8.0 Hz, 12-H), 3.84 (1H, t, *J* = 11.0 Hz, H-2 *endo*), 3.20–3.14 (1H, m, H-1 *endo*), 3.12–2.97 (5H, m, H-1 *exo*, 2 × H-9, 2 × H-10), 2.83 (1H, ddd, *J* = 12.0, 5.5, 3.7 Hz, H-2 *exo*), 1.17 (9H, s, *t*-Bu); δ_C_ (125 MHz, CDCl_3_) 145.7 (C), 144.4 (C), 139.9 (C), 139.4 (C), 139.1 (C), 134.2 (CH), 133.5 (CH), 133.0 (CH), 132.9 (CH), 132.8 (CH), 132.5 (CH), 131.0 (CH), 46.3 (C), 35.4 (CH_2_), 35.4 (CH_2_), 34.8 (CH_2_), 34.7 (CH_2_), 30.9 (CH_3_); *m/z* (EI+) 296 [M]^+^, 240 [M – *t*-Bu]^+^, 207 [M − S*t*-Bu]^+^, 136, 104, 91, 78 (Found: [M]^+^, 296.1591. C_20_H_24_S requires [M]^+^, 296.1593).

### (*S*_p_)-(+)-Thioacetic acid *S*-[2.2]paracyclophan-4-yl ester [(*S*_p_)-**7**]

Boron tribromide (0.36 mL, 3.77 mmol, 1.1 equiv) was added to a solution of (*S*_p_)-(+)-4-*t*-butylsulfanyl[2.2]paracyclophane (*S*_p_)-**6** (1.0 g, 3.43 mmol, 1.0 equiv) and acetyl chloride (1.7 mL, 24.01 mmol, 7.0 equiv) in toluene (34.3 mL) at rt. The reaction was stirred for 1 hour then poured into a solution of ice cold saturated aqueous NH_4_Cl (200 mL). Aqueous phase was extracted with Et_2_O (3 × 50 mL). The combined organic phase was washed with aqueous Na_2_S_2_O_3_ (10% w/v) (3 × 50 ml), dried (MgSO_4_) and concentrated. Purification by chromatography (10% Et_2_O/hexane) afforded (*S*_p_)-**7** as a white solid (0.53 g, 55%); mp = 134–136 °C; [α]_D_ + 84.3 (*c* 1, CHCl_3_) (assumed 80% *ee* see text); ν_max_ (film) 2920, 2850, 1694, 1498, 1476, 1447, 1432, 1408, 1113, 954, 906, 850 and 792 cm^−1^; δ_H_ (500 MHz, CDCl_3_) 6.68 (1H, d, *J* = 8.0 Hz, H-13), 6.60 (2H, s, H-15, H-16), 6.52 (3H, m, H-5, H-7, H-8), 6.42 (1H, d, *J* = 7.5 Hz, H-12), 3.41–3.35 (1H, m, H-2 *endo*), 3.13–2.97 (5H, m, H-1 *endo*, 2 × H-9, 2 × H-10), 2.99 (1H, t, *J* = 10.2 Hz, H-1 *exo*), 2.92–2.86 (1H, m, H-2 *exo*), 2.36 (3H, s, Me); δ_C_ (125 MHz, CDCl_3_) 190.7 (CO), 143.1 (C), 140.4 (C), 139.4 (C), 139.1 (C), 135.1 (CH), 135.0 (CH), 134.5 (CH), 133.2 (CH), 133.1 (CH), 132.3 (CH), 130.2 (CH), 129.2 (CH), 35.4 (CH_2_), 34.9 (CH_2_), 34.6 (CH_2_), 34.4 (CH_2_), 30.2 (CH_3_); *m/z* (EI+) 282 [M]^+^, 240 [M+H-Ac]^+^, 207 [[2.2]paracyclophane]^+^, 178, 136, 104, 91, 78, 43 (Found: [M]^+^, 282.1075. C_18_H_18_OS requires [M]^+^, 282.1073).

### (*S*_p_)-(+)-[2.2]Paracyclophane-4-thiol [(*S*_p_)-**2**]

To a solution of (*S*_p_)-**7** (0.51 g, 1.80 mmol, 1.0 equiv) in methanol (18 mL) was added K_2_CO_3_ (3.71 g, 26.84 mmol, 15 equiv) and the reaction stirred for 3 hours at rt. The reaction was poured into saturated aqueous NH_4_Cl solution (100 mL) and extracted with Et_2_O (3 × 100 mL). The combined organic layers were dried (MgSO_4_) and concentrated before purification by chromatography (1% Et_2_O/hexane) gave (*S*_p_)-**2** as a white solid (0.14 g, 32%); mp = 144–146 °C; [α]_D_ + 164.0 (*c* 1, CHCl_3_) (assumed 80% *ee* see text); ν_max_ (film) 3011, 2929, 2848, 2558, 1587, 1548, 1499, 1480, 1449, 1432, 1411, 1060, 938, 897, 849, 804 and 791 cm^−1^; δ_H_ (500 MHz, CDCl_3_) 7.21 (1H, d, *J* = 7.5 Hz, H-13), 6.57 (1H, d, *J* = 7.5 Hz, H-8), 6.47 (1H, d, *J* = 7.5 Hz, H-7), 6.43 (1H, d, *J* = 7.5 Hz, H-12), 6.40 (2H, t, *J* = 7.5 Hz, H-15, H-16), 6.22 (1H, s, H-5), 3.41 (1H, t, *J* = 12.0 Hz, H-2 *endo*), 3.26 (1H, ddd, J = 13.0, 6.0, 3.9 Hz, H-1 *endo*), 3.13 (1H, s, S-H), 3.11–3.01 (4H, m, 2 × H-9, 2 × H-10), 2.90–2.87 (1H, t, *J* = 9.0 Hz, H-1 *exo*), 2.83–2.77 (1H, m, H-2 *exo*); δ_C_ (125 MHz, CDCl_3_) 140.4 (C), 139.3 (C), 139.1 (C), 138.5 (C), 135.8 (CH), 134.8 (CH), 133.4 (CH), 132.8 (CH), 131.9 (CH), 131.5 (CH), 130.5 (CH), 127.7 (CH), 35.4 (CH_2_), 34.9 (CH_2_), 34.6 (CH_2_), 33.1 (CH_2_); *m/z* (EI+) 240 [M]^+^, 207 [[2.2]paracyclophane]^+^, 136, 104, 91, 78 (Found: [M]^+^, 240.0969. C_16_H_16_S requires [M]^+^, 240.0967).

### (*R*_p_,*R*_S_)-(–)-4-*tert*-Butylsulfinyl-5-amino[2.2]paracyclophane [(*R*_p_,*R*_S_)-**8**]

To a solution of (*R*_p_,*R*_S_)-(–)-4-*t*-butylsulfinyl[2.2]paracyclophane [(*R*_p_,*R*_S_)-**5**] (5.80 g, 18.6 mmol, 1.0 equiv) in THF (350 mL) at 0 °C was added *n*-BuLi (2.5M in hexanes; 16.5 mL, 41.25 mmol, 2.2 equiv) dropwise over 30 min to give an orange solution. After 1 h tosyl azide (9.20 g, 46.70 mmol, 2.5 equiv) was added and the reaction warmed to rt over 18 h. NaBH_4_ (6.45 g, 171 mmol, 9 equiv) and *tetra-n*-butyl ammonium iodide (6.31 g, 17.1 mmol, 0.9 equiv) were added and the reaction stirred for a further 24 h at rt whereupon a further portion of NaBH_4_ (2.80 g, 74.0 mmol, 4.0 equiv) was added. After further stirring at rt for 5 days the reaction was poured into saturated aqueous NH_4_Cl (250 mL) causing effervescence. The aqueous phase was extracted with Et_2_O (500 mL + 200 mL) and the combined organic extracts dried (MgSO_4_) and the solvent removed. The residue was purified by chromatography (neutralized silica gel 40% Et_2_O/*n*-heptane) to yield **8** as a pale yellow solid powder, which was recrystalised from CHCl_3_ / heptane (4.00 g, 66%); mp = 130–132 °C; [α]_D_ −118.0 (*c* 1, CHCl_3_) (assumed 80% *ee* see text); ν_max_ (film) 3430, 3055, 2987, 1637, 1421, 1265, 896, 739 and 705 cm^−1^; δ_H_ (500 MHz, CDCl_3_) 7.15 (1H, d, *J* = 8.0 Hz, H-13), 6.89 (1H, d, *J* = 7.5 Hz, H-16), 6.63 (1H, d, *J* = 8.0 Hz, H-15), 6.42 (1H, d, *J* = 8.0 Hz, H-7), 6.35 (1H, d, *J* = 7.5 Hz, H-12), 6.01 (1H, d, *J* = 7.5 Hz, H-8), 5.62 (2H, s, broad, NH_2_), 3.46 (1H, t, *J* = 12.0 Hz, H-2 *endo*), 3.23–3.18 (1H, m, H-1 *endo*), 3.12–3.01 (4H, m, H-1 *exo*, H-9 *endo*, 2 × H-10), 2.71–2.62 (2H, m, H-2 *exo*, H-9 *exo*), 1.19 (9H, s, *t-*Bu); δ_C_ (125 MHz, CDCl_3_) 150.6 (C), 141.6 (C), 138.5 (C), 138.1 (C), 133.1 (C), 132.1 (C), 131.9 (CH), 129.7 (CH), 127.1 (CH), 126.5 (CH), 123.0 (CH), 114.7 (CH), 60.19 (C), 34.1 (CH_2_), 33.9 (CH_2_), 32.8 (CH_2_), 30.8 (CH_2_), 23.6 (CH_3_); *m/z* (ESI+) 350.1547 [M+Na]^+^ (Found: [M+Na]^+^, 350.1549. C_20_H_25_OSNNa requires [M+Na]^+^, 350.1546).

### (*R*_p_)-(–)-4-*tert*-Butylsulfanyl-5-amino[2.2]paracyclophane [(*R*_p_)-**9**]

Triethylamine (1.47 mL, 10.53 mmol, 10 equiv) followed by trichlorosilane (2.60 mL, 25.76 mmol, 15.0 equiv) were added carefully to a solution of (*R*_p_,*R*_S_)-4-(–)-*t*-butylsulfinyl-5-amino[2.2]paracyclophane [(*R*_p_,*R*_S_)-**8**] (0.50 g, 1.61 mmol, 1.0 equiv) in toluene (8 mL,) at 0 °C and the reaction heated to reflux for 16 h. The reaction was cooled to 0 °C and aqueous NaOH (3M; 100 mL) was added. The aqueous phase was extracted with Et_2_O (3 × 50 mL), dried (MgSO_4_) and concentrated to give a pale yellow solid. Purification by chromatography (neutralized silica, 20% Et_2_O/hexane) gave (*R*_p_)-**9** as a pale yellow solid (0.19 g, 40%); mp = 72–74 °C; [α]_D_ −192.3 (*c* 1, CHCl_3_) (assumed 80% *ee* see text); ν_max_ (film) 3472, 3364, 2931, 2856, 1592, 1464, 1456, 1429, 1408, 1155, 876, 802, 741 and 717 cm^−1^; δ_H_ (500 MHz, CDCl_3_) 7.0 (1H, d, *J* = 9.5 Hz, H-13), 6.60 (1H, d, *J* = 10.0 Hz, H-7), 6.47 (1H, d, *J* = 10.0 Hz, H-12), 6.41 (1H, d, *J* = 9.5 Hz, H-8), 6.33 (1H, d, *J* = 9.5 Hz, H-15), 6.23 (1H, d, *J* = 9.5 Hz, H-16), 4.47 (2H, br s, NH_2_), 3.70 (1H, ddd, *J* = 14.0, 9.6, 3.2 Hz, H-2 *endo*), 3.11–2.96 (5H, m, H-1 *endo*, 2 × H-9, 2 × H-10), 2.78–2.65 (2H, m, H-1 *exo*, H-2 *exo*), 1.21 (9H, s, *t*-Bu); δ_C_ (125 MHz, CDCl_3_) 149.4 (C), 147.3 (C), 139.1 (C), 138.3 (C), 135.7 (CH), 133.0 (CH), 132.2 (CH), 129.7 (CH), 126.3 (CH), 124.3 (C), 123.0 (CH), 117.9 (C), 48.1 (C), 35.3 (CH_2_), 34.1 (CH_2_), 32.8 (CH_2_), 32.3 (CH_2_), 30.9 (CH_3_); *m/z* (EI+) 311 [M]^+^, 255 [M+H−*t*-Bu]^+^, 207 [[2.2]paracyclophane]^+^, 151, 106 (Found: [M+H]^+^, 312.1791. C_20_H_26_NS requires [M+H]^+^, 312.1780).

### (*R*_p_)-(+)-[2.2](4,7)Benzo[*d*]thiazoloparacyclophane [(*R*_p_)-**10**]

To a solution of **9** (141 mg, 0.45 mmol, 1.0 equiv) and paraformaldehyde (54.5 mg, 1.82 mmol, 2.0 equiv) in dioxane (5.0 mL) and water (1.0 mL) was added aqueous HCl_conc._ (9.0 M; 0.108 mL). Pyridine (0.19 mL, 1.82 mmol, 2.0 equiv) was added and the reaction mixture heated to reflux for 48 h. The reaction mixture was poured into aqueous NaOH (3.0 M; 20 mL) and the aqueous phase extracted with Et_2_O (3 × 30 mL). The combined organic phases were dried (MgSO_4_) and the solvent removed; purification by chromatography (neutralized silica gel, 50% Et_2_O/heptane) furnished (*R*_p_)-**10** as a pale yellow powder (36.3 mg, 28%); mp = 144–146 °C; [α]_D_ +67.4 (*c* 1, CHCl_3_) (assumed 80% *ee* see text); ν_max_ (film) 3467, 3005, 2978, 2934, 2873, 1448, 1384, 1351, 1217 and 1111 cm^−1^; δ_H_ (500 MHz, CDCl_3_; paracyclophane numbering) 8.89 (1H, s, thiazole CH), 6.81 (1H, d, *J* = 7.5 Hz, H-13), 6.68 (1H, d, *J* = 7.5 Hz, H-12), 6.52 (1H, d, *J* = 8.0 Hz, H-16), 6.46 (1H, d, *J* = 7.5 Hz, H-15), 6.18 (1H, d, *J* = 7.5 Hz, H-7), 5.85 (1H, d, *J* = 7.5 Hz, H-8), 3.99–3.93 (1H, m, H-2 *endo*), 3.27–3.21 (1H, m, H-1 *endo*), 3.17–3.11 (1H, m, H-9 *endo*), 3.06–2.99 (5H, m, H-1 *exo*, H-2 *exo*, H-9 *exo*, 2 × H-10); δ_C_ (125 MHz, CDCl_3_) 154.4 (CH), 151.8 (C), 139.2 (C), 137.2 (C), 134.8 (C), 134.4 (C), 132.8 (C), 132.3 (CH), 131.7 (CH), 130.5 (CH), 126.2 (CH), 124.9 (CH), 35.0 (CH_2_), 34.5 (CH_2_), 33.5 (CH_2_), 32.4 (CH_2_); *m/z* (EI+) 265 [M]^+^, 161, 104, 78 (Found: [M]^+^, 265.0921. C_17_H_15_NS requires [M]^+^, 265.0920).
